# Astrocyte-specific overexpressed gene signatures in response to methamphetamine exposure in vitro

**DOI:** 10.1186/s12974-017-0825-6

**Published:** 2017-03-09

**Authors:** Nikki Bortell, Liana Basova, Svetlana Semenova, Howard S. Fox, Timothy Ravasi, Maria Cecilia G. Marcondes

**Affiliations:** 1 Cellular and Molecular Neurosciences Department, The Scripps Research Institute, La Jolla, CA 92037 USA; 2 Department of Psychiatry, University of California San Diego, San Diego, CA 92093 USA; 3 Department of Experimental Pharmacology, University of Nebraska Medical School, Omaha, NE 68198 USA; 4 KAUST Environmental Epigenetic Program (KEEP), Division of Biological and Environmental Sciences and Engineering, King Abdullah University of Science and Technology, Thuwal, 23955 Kingdom of Saudi Arabia; 5 Department of Medicine, Division of Genetic, University of California San Diego, 9500 Gilman Drive, La Jolla, California 92093 USA; 6 Anschutz Medical Campus, University of Colorado, Denver, CO USA; 7 Present address: San Diego Biomedical Research Institute, 10865 Road to the Cure, Suite 100 - San Diego, San Diego, CA 92121 USA

**Keywords:** Astrocytes, Methamphetamine, Central nervous system, Systems biology

## Abstract

**Background:**

Astrocyte activation is one of the earliest findings in the brain of methamphetamine (Meth) abusers. Our goal in this study was to identify the characteristics of the astrocytic acute response to the drug, which may be critical in pathogenic outcomes secondary to the use.

**Methods:**

We developed an integrated analysis of gene expression data to study the acute gene changes caused by the direct exposure to Meth treatment of astrocytes in vitro, and to better understand how astrocytes respond, what are the early molecular markers associated with this response. We examined the literature in search of similar changes in gene signatures that are found in central nervous system disorders.

**Results:**

We identified overexpressed gene networks represented by genes of an inflammatory and immune nature and that are implicated in neuroactive ligand-receptor interactions. The overexpressed networks are linked to molecules that were highly upregulated in astrocytes by all doses of methamphetamine tested and that could play a role in the central nervous system. The strongest overexpressed signatures were the upregulation of MAP2K5, GPR65, and CXCL5, and the gene networks individually associated with these molecules. Pathway analysis revealed that these networks are involved both in neuroprotection and in neuropathology. We have validated several targets associated to these genes.

**Conclusions:**

Gene signatures for the astrocytic response to Meth were identified among the upregulated gene pool, using an in vitro system. The identified markers may participate in dysfunctions of the central nervous system but could also provide acute protection to the drug exposure. Further in vivo studies are necessary to establish the role of these gene networks in drug abuse pathogenesis.

**Electronic supplementary material:**

The online version of this article (doi:10.1186/s12974-017-0825-6) contains supplementary material, which is available to authorized users.

## Background

Astrocytes are glial cells that are involved in numerous brain functions, including interacting with neurons and maintaining brain structure [1, 2], synthesizing cholesterol [3], and controlling synaptogenesis and neuronal plasticity [4–6]. In addition, together with microglia cells, astrocytes are first responders to both systemic and central nervous system (CNS) localized insults [7, 8]. These cells can initiate an inflammatory response, which can interestingly both promote tissue healing and neuronal loss and damage, as well as control the permeability of the blood-brain barrier (BBB) [9–11].

Drug abuse is one of the factors that can induce the activation of astrocytes [12]. One such drug is methamphetamine (Meth), which is widely used due to its strong effects as a psychotropic stimulant and its low price. Meth abusers develop an enormous number of degenerative symptoms, particularly in the CNS, manifested by cognitive deficits and motor dysfunction [13]. Astrocytic activation is one of the most common findings in the brain of Meth abusers, as well as in various models of Meth exposure [14–17], often associated with neurotoxicity. The effects of Meth on astrocytes could be potentially direct and have been attributed to its binding to sigma1 receptors [18].

In the present studies, we tested the hypothesis that astrocytes, as important first responders, may develop phenotypic changes that can contribute and be associated to neurological decline and multiple disorders commonly found in Meth abusers. We evaluated whether Meth can affect astrocytes by producing changes in gene expression and whether such changes can be critical attributes in the development of CNS disorders. We examined genes that were upregulated following Meth exposure, with an in vitro approach using primary cortical astrocytic cultures. Using systems biology, we have integrated the expression changes caused by Meth and have identified important astrocytic patterns, fingerprints, and pathways among genes that are transcriptionally enriched by the exposure to Meth. Although the analysis is limited to upregulated genes, these changes could have important implications in the development of neurological symptoms commonly associated with drug abuse.

## Methods

### Primary rat astrocyte cultures

E18 embryonic Sprague Dawley rat cortical astrocytes (BrainBits LLC, Springfield, IL) were cultured on poly-D-lysine-coated coverslips and were maintained in Neuro basal medium (Invitrogen, Carlsbad, CA) with 10% horse serum and 3 mM glutamine (Invitrogen), until confluence was reached (day 12).

### Methamphetamine treatment

(+)-Methamphetamine hydrochloride (Sigma-Aldrich, Saint Louis, MO) was added to the confluent astrocytic cultures, at the final concentrations of 1, 10, and 100 μM, diluted in PBS. The drug was maintained in the cultures for 24 h prior to the harvesting of the cells. Control cultures were incubated with PBS vehicle. All results derive from three independent experiments, each one performed in duplicate.

### Apoptosis detection

The rat astrocytes were tested for development of apoptosis 24 h after Meth treatment, using the terminal deoxynucleotidyl transferase dUTP (TdT) in situ TACS Blue (R&D systems), following the manufacturer’s instructions. Counterstaining was performed with Gill’s hematoxylin (Sigma-Aldrich, St. Louis, MO). Coverslips were applied over Cytoseal 60 mounting media (EMS, Hatfield, PA), and cells were inspected in light microscope.

### Cell harvest and RNA extraction

Total RNA was isolated from the cells using Trizol reagent (Thermofisher, Waltham, MA), according to the manufacturer’s instructions. Total RNA concentration was measured using the Nanodrop spectrophotometer and then used for reverse transcription and for gene array (below).

### Gene expression array

The integrity of total RNAs was examined in an Agilent Bioanalyzer 2100 (Agilent Technologies, Santa Clara, CA, USA). Total RNA concentration was measured using the Nanodrop spectrophotometer. The mouse Agilent microarray service was performed by Phalanx Biotech (San Diego, CA). A total of 4 μg Cy5-labeled RNA targets were hybridized to Gene Expression v2 4x44K Microarrays (Agilent Technologies, Santa Clara, CA), according to the manufacturer’s protocol. The data were analyzed using the provided manufacturer’s protocol. Following the hybridization, fluorescent signals were scanned using an Axon 4000 (Molecular Devices, Sunnyvale, CA, USA). Three replicates per condition were used. Microarray signal intensity of each spot was analyzed using the GenePix 4.1 software (Molecular Devices, Sunnyvale, CA, USA). Each signal value was normalized using the R program in the limma linear models package (Bioconductor 3.2, https://bioconductor.org).

### Gene expression analysis

Raw data was loaded into ArrayStudio (Omicsoft Corporation, Cary, NC) and first filtered based on a built-in ANOVA, as well as a *t* test, applied to fold changes between experimental and control conditions. Significant changes had a *p* value <0.05. In addition, maximum least-squares (Max LS) mean ≥ 6 and a false discovery rate by the Benjamini-Hochberg correction (FDR_BH) <0.01 were applied. Using this method, we found many genes with raw *p* values <0.05, but if the FDR_BH did not reach <0.01, they were discarded. In this particular analysis set, the genes were further filtered to express a robust fold change above 4 between control and experimental cultures. These filters allowed the identification of significant, above background gene expression changes. The list of genes that were significantly upregulated by Meth in astrocytes, following the described criteria, were loaded into Cytoscape 3.3 (http://cytoscape.org), using GeneMania [19], to identify significantly changed interaction networks of genes and relevant pathways. Pathway enrichment was examined using iPathwayGuide (Advaita Bioinformatics, Plymouth, MI) platform [20] and DAVID Bioinformatics database [21] (https://david.ncifcrf.gov), which utilize the Kyoto Encyclopedia of Genes and Genomes (KEGG) (www.genome.jp/kegg) and in Gene Ontology (GO) terms (http://geneontology.org/page/go-enrichment-analysis). Diseases associated to the changed profiles and genes were identified in KEGG.

### RT-PCR

Validation of gene array data was performed both in the same samples and in two additional independent experiments. RNA was reverse transcribed using SuperScript III Reverse Transcriptase (Invitrogen, Waltham, MA). Most primers were purchased from Qiagen (Valencia, CA). PCRs were performed using RT^2^ SYBR Green ROX FAST Mastermix (Qiagen), in a 7900HT Fast Real-Time PCR System with Fast 96-Well Block Module (Applied Biosystems, Foster City, CA) with a SDS Plate utility v2.2 software (Applied Biosystems). The results were normalized to the expression of GAPDH.

### Protein extraction and western blots

Following a wash with ice-cold PBS, protein from cell cultures was extracted by lysis in radio-immunoprecipitation assay buffer (RIPA—Thermo Fisher Scientific, Waltham, MA) in the presence of Complete protease inhibitor cocktail tablets (Roche Molecular Biochemicals, Indianapolis, IN). The cells were scraped and transferred to a microfuge tube and span at 10,000 rpm at 4 °C for 10 min. The supernatant was transferred to a new tube and protein concentration was measured using a Bradford Reagent (BioRad, Hercules, CA). Protein was stored in −20 °C until use. Ten micrograms of protein were loaded into each lane of SDS-PAGE electrophoresis gels (BioRad) in 4–20% gradient gels under reducing conditions. Transfer and immunodetection were performed as previously described [22]. Nonspecific antibody binding was blocked using 5% nonfat dried milk for 1 h at room temperature. Immunoblotting was carried out with antibodies against MEK5/MAP2K5 (PA5-29236, Thermo Fisher Scientific), TDAG8/GPR65 (BS-7668R, Bioss, Inc./VWR, Radnor, PA), and b-actin (Cell Signaling, Danvers, MA), followed by secondary antibody HRP-conjugated anti-rabbit IgG (GE Healthcare, Little Chalfont, UK). Blots were developed in film (Kodak) with 1:1 solution of Super Signal West Pico Chemiluminescent Substrate and Luminol/Enhancer (Thermo Fisher Scientific, Rockford, IL). Bands were scanned and band intensities were calculated in ImageJ 1.43u (National Institute of Health, Bethesda, MD). Experimental bands were normalized to the intensity of b-actin bands in each sample.

### Immunocytochemistry

Cells were cultured on poly-L-lysine (Sigma-Aldrich)-treated 8-well glass chamber slides (Thermo Scientific), fixed with 4% paraformaldehyde for 20 min in the dark, and then washed with PBS. Wells were then incubated with PBS containing 0.1% Triton X-100 for 15 min at room temperature, rinsed 3 times with PBS, and then blocked with 5 g/l Casein (Sigma-Aldrich) in PBS, containing 0.5 g/l Thimerosal (Sigma-Aldrich) for 1 h at room temperature. The primary antibodies against MEK5/MAP2K5 (PA5-29236, Thermo Fisher Scientific), TDAG8/GPR65 (BS-7668R, Bioss, Inc./VWR, Radnor, PA), and CXCL5 (BS-2549R, Bioss Inc./VWR, Radnor, PA) were diluted in blocking solution and placed in the wells for 2 h at room temperature. Then, cells were rinsed 3 times for 10 min with 1% blocking solution in PBS, followed by incubation with a secondary Alexa594-labeled donkey anti-rabbit IgG (Thermo Fisher Scientific) for 2 h at room temperature, in the dark. After rinsing, 4′,6-diamidino-2-phenylindole dihydrochloride (DAPI) was diluted to 300 ng/ml in 1% blocking solution for 10 min, in the dark. Cells were rinsed and maintained in PBS and observed in a Nikon A1R laser-scanning confocal mounted onto a Nikon-inverted Ti-E scope (Nikon, Melville, NY), and with a 20× PlanApo objective, 0.8NA (Nikon) and images were acquired using a NIS-Elements C software (Nikon). Fluorescence intensity was normalized against background (secondary antibody only) and calculated in ImageJ 1.43u (National Institute of Health, Bethesda, MD).

### Statistical analysis

Group comparisons for individual genes across different culture conditions were performed using one-way ANOVA, followed by Bonferroni’s post hoc tests. The difference between the means was considered significant at *p* < 0.05. Tests were performed using Prism software (GraphPad Software, San Diego, CA, USA) for Macintosh.

## Results

Astrogliosis is among the earliest consequences of Meth use to the CNS. We investigated the early effects of Meth exposure on primary cortical astrocytes, by focusing on genes that were upregulated to above a conservative 4-fold threshold, which corresponded to more than 62% of the total changes in gene expression caused by the drug at any concentration over 24 h of exposure, as determined by gene array. Importantly, over the course of the experiment, we did not observe significant decrease of cell viability due to the exposure to different concentrations of Meth, as assessed by TdT detection by in situ hybridization (not shown).

**Fig. 1:**
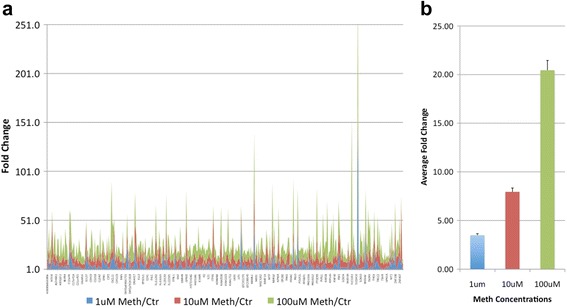
Changes in astrocytic gene expression pattern following exposure to different doses of Meth for 24 h. **a** Three doses of Meth were used, 1 μM (*blue diamonds*), 10 μM (*red squares*), and 100 μM (*green triangles*). The genes that showed a robust 4-fold increase in all Meth treatments were plotted as fold change in Meth-treated cells compared to controls. **b** The average fold change of all the genes was plotted for each one of the doses of Meth, and the Pearson coefficient was calculated (*r* ^2^ = 0.89). One-way ANOVA *p* value ≤0.0001

We found that 411 genes were increased when the astrocytic cultures were exposed to Meth at concentrations of 10 or 100 μM, in comparison to controls, while in treatments with 1 μM of Meth, 180 genes were significantly increased. All together, 179 genes were significantly upregulated to above 4-fold by all the three doses of Meth compared to vehicle-treated astrocytes (Table 1). The correlation coefficient confirms that for the majority of the genes, Meth induced a dose-dependent response pattern (Table 1), which is visualized in Fig. 1. Figure 1a shows fold change of all the upregulated genes at the different doses of Meth. All genes that increased to above 4-fold at 10 μM of Meth compared to control were also significantly increased with 100 μM, but only 32% of those were also increased by the 1-μM condition. The calculation of the average fold change showed a significant overall dose-response effect (Fig. 1b), with a Pearson coefficient equal to 0.89 and a *p* value ≤0.0001. However, the examination of a correlation coefficient in all the individual upregulated genes showed that 28% of all genes exhibited a flat response that was equal in all 3 doses, while 22% of the genes showed a negative correlation coefficient, suggesting an inverse dose-response effect.

**Table 1:** List of genes that were significantly upregulated to above fourfold compared to control, in astrocytic cultures stimulated with 1, 10 or 100 μM of Meth for 24 h and the calculated correlation coefficient

Genes	1 μM Meth/Ctr	10 μM Meth/Ctr	100 μM Meth/Ctr	Correlation coefficient
HRH4	5.22	6.00	14.15	0.999994447
PRO2610	5.96	7.26	22.63	0.999923265
LOC160313	7.61	8.69	21.71	0.999909473
LOC284244	4.31	4.68	7.68	0.999847888
IL2RG	9.46	10.02	13.82	0.99936847
FLJ21125	4.64	4.92	6.73	0.999151048
CXCL11	10.63	12.83	25.68	0.998567259
CFLAR	4.03	4.05	4.81	0.998443584
ZNF407	14.94	15.12	23.88	0.997905826
DLEU2	4.42	4.65	27.26	0.997318927
BLZF1	5.86	6.02	30.54	0.997077518
CA5A	4.35	7.04	20.25	0.997050779
WNT4	9.36	9.28	17.78	0.99591735
ZNF256	5.47	7.97	18.80	0.995466993
FLJ23022	5.35	5.26	10.05	0.995146093
ANKRD2	7.26	9.06	15.97	0.99339194
RPIB9	8.30	7.64	16.58	0.988926108
EIF5A2	8.95	9.24	10.13	0.988863198
NAV3	18.93	25.26	43.44	0.985779001
CXCL11	12.21	5.50	72.41	0.984990642
LOC115648	6.34	5.73	11.47	0.984194119
EPOR	5.35	5.05	7.68	0.982893395
FHL5	6.31	5.27	14.20	0.98214045
FLJ12476	5.03	4.14	11.13	0.980241319
AGTR2	4.51	10.44	25.06	0.979647991
PTPN11	5.92	5.39	9.24	0.978342528
ERBB3	4.21	5.69	9.13	0.976733781
C17orf31	7.89	9.07	11.75	0.975527055
COVA1	4.97	5.76	7.51	0.97455129
IL21	6.43	5.35	12.13	0.973672943
MGC5347	7.52	5.04	19.93	0.971790582
PRO1483	10.46	11.72	14.24	0.968741946
IL1RN	5.88	6.42	7.44	0.965964206
ROCK1	5.21	8.71	15.25	0.964364225
ZCCHC4	5.85	4.36	11.88	0.96376171
HPCA	4.04	5.48	7.90	0.956547336
NUP62	5.45	4.72	7.97	0.955960582
RW1	19.68	14.85	35.53	0.953075462
C14orf105	13.55	4.01	38.76	0.938912869
CXCL5	11.65	4.91	27.58	0.93015087
IL18RAP	7.38	6.14	10.30	0.929932455
ZNF154	5.31	4.37	7.48	0.928233605
CPB1	7.73	4.35	14.93	0.92105335
ABCC3	13.96	12.93	16.14	0.920806618
C10orf6	7.30	5.12	11.75	0.916688112
UPK1A	4.89	5.10	5.34	0.914921573
PTGER3	6.96	27.56	49.18	0.910059159
HYAL3	6.44	4.78	9.16	0.893055409
RANBP2L1	7.02	6.85	7.31	0.892766347
IGF1R	8.99	8.40	9.89	0.883899673
PRKRIP1	7.88	7.09	9.06	0.88130575
CPT1B	5.01	4.01	6.43	0.873310822
CDH19	4.56	7.24	9.34	0.871735485
FLJ20130	5.40	5.01	5.93	0.867880389
ACSL6	6.38	11.00	14.22	0.855571102
ALS2CR8	10.31	5.58	15.43	0.834990904
PHF3	30.59	15.06	47.05	0.831376032
TRGC2	8.13	20.42	26.81	0.812813175
POU4F1	4.38	8.73	10.68	0.791279314
C14orf136	10.74	8.45	12.60	0.787208769
MAP2K5	47.30	40.66	51.74	0.751719778
FLJ10254	13.09	4.71	18.20	0.734959034
KIAA0125	6.26	11.21	12.42	0.711285891
MAN1A2	4.45	7.58	8.29	0.702683293
FLJ21463	18.35	28.81	30.81	0.686425762
ED1	28.51	18.13	32.47	0.653331221
C21orf55	4.04	5.13	5.26	0.646315568
GNRHR	4.46	4.91	4.95	0.6183026
ADCK4	8.69	11.02	11.14	0.602818956
NSBP1	12.62	25.17	25.78	0.602619958
SSBP1	6.90	10.61	10.76	0.596964796
PPFIBP1	5.71	4.16	6.10	0.590195955
TRIM29	4.78	19.48	19.47	0.569146877
CYP2C9	9.23	9.13	9.25	0.56683014
OR2B2	7.57	8.57	8.56	0.561685078
PRELP	6.43	5.61	6.51	0.504686246
SSA2	37.28	4.89	39.64	0.481229307
PRO1600	6.99	12.36	11.48	0.438516158
XCL1	19.84	8.61	19.69	0.416074655
HSD11B1	10.34	7.07	10.24	0.403740827
CYP19A1	7.65	5.86	7.50	0.358844363
MGC3262	8.68	4.67	8.30	0.347905863
LAK	6.66	4.17	6.39	0.335763779
FLJ11292	15.84	26.79	23.78	0.329911393
KIAA0506	4.56	34.32	25.68	0.314405937
DNAH17	4.47	6.52	5.90	0.302712912
SLC28A3	7.41	17.06	14.14	0.301973863
ELSPBP1	7.95	11.66	10.44	0.274977991
ZNF41	4.86	5.39	5.20	0.241177993
FLJ11348	8.42	11.42	10.30	0.226653077
EPB41	11.15	7.87	10.37	0.208972082
SMG1	154.71	11.40	120.32	0.207904327
TNP2	23.46	15.58	21.25	0.164160589
GAGE8	4.48	13.21	9.39	0.153207816
CENPF	4.21	5.08	4.67	0.116060881
PIGO	4.46	10.82	7.73	0.09831086
NFAT5	23.00	6.44	17.20	0.088932883
POLR2A	9.09	4.73	7.55	0.0851526
CLCA1	5.32	12.11	8.72	0.082447917
PAX2	9.46	5.85	8.16	0.079652497
GPR65	15.67	38.31	26.87	0.075789877
FLJ13265	6.84	20.68	13.46	0.057111998
MSR1	7.69	4.99	6.58	0.018886308
POGZ	42.73	12.20	29.02	−0.023536687
CPB2	8.02	4.16	6.27	−0.02790024
MAP3K2	4.82	5.09	4.93	−0.032387929
HCFC1	6.05	12.51	8.24	−0.100973451
CG018	6.96	16.00	9.89	−0.117326039
VPS13D	4.60	6.27	5.10	−0.144143951
ZNF198	25.51	20.32	22.63	−0.145242887
TBXA2R	4.72	6.30	5.19	−0.150283188
ICOSL	5.22	12.20	7.06	−0.183375741
KIAA1111	7.10	10.54	7.92	−0.211199313
LTBP1	9.46	7.01	7.95	−0.211941907
GLI2	4.23	7.75	5.06	−0.212825862
MR1	8.98	20.62	11.69	−0.215175838
FLJ22349	5.58	4.14	4.66	−0.237602369
FLJ14075	11.93	8.12	9.50	−0.238880951
ATF5	5.48	4.41	4.78	−0.259393542
SLC38A3	4.74	10.60	5.68	−0.286870158
C13orf10	26.12	16.12	19.26	−0.289504282
DKFZp547G183	27.90	12.34	17.11	−0.297276605
FLJ13315	4.10	14.76	5.39	−0.324025634
LAD1	4.16	5.80	4.35	−0.325934689
CXCL10	5.56	10.82	6.16	−0.330535675
HS3ST1	10.35	6.53	7.46	−0.363755006
PPARBP	13.49	4.11	6.08	−0.39467915
XTP2	8.78	4.50	5.39	−0.395501804
CCNF	7.88	5.06	5.63	−0.40201752
KCNJ5	6.48	8.95	6.50	−0.422109284
EPS15L1	5.88	17.45	5.74	−0.437001047
MDM2	17.42	6.02	7.88	−0.437851968
NDRG2	4.12	10.59	4.02	−0.439445915
PC	7.00	19.67	6.18	−0.475353048
KRTAP2-2	7.24	20.52	5.99	−0.495867331
TP73L	7.46	4.80	5.04	−0.49958314
PYGO1	6.10	4.07	4.24	−0.50597972
ZNF492	12.76	53.53	8.28	−0.506554374
EIF3S5	7.75	32.72	4.24	−0.526505038
KCNN2	6.32	4.28	4.39	−0.527171032
KIAA1061	10.24	4.60	4.64	−0.564918257
ICAM5	5.65	10.15	4.69	−0.569881474
ADAM3A	13.97	39.21	7.92	−0.584876673
KBTBD10	13.67	46.06	5.78	−0.586881473
MLL4	8.09	20.74	4.94	−0.589848953
C14orf161	7.61	5.92	5.76	−0.632312086
LOC51233	5.49	8.92	4.27	−0.642741323
THEA	8.13	12.93	6.35	−0.648630838
LOC153077	49.55	10.43	5.54	−0.649861448
RNF40	7.17	4.47	4.10	−0.655819595
SAMSN1	12.07	25.74	6.02	−0.67845578
MYO15B	15.81	10.96	10.02	−0.687237714
MPHOSPH9	7.66	4.97	4.38	−0.699214766
GAD2	19.96	9.83	6.74	−0.738789688
DKFZp761K1824	16.25	7.33	4.25	−0.7549633
ERO1LB	17.38	9.75	6.47	−0.785862655
CDC27	7.96	11.36	4.92	−0.802905584
R3HDM	9.32	13.58	5.26	−0.814306845
FER1L4	4.37	4.69	4.05	−0.819705072
CRISP1	11.33	17.94	4.26	−0.83259712
KIAA0711	12.39	10.16	8.77	−0.839558326
SHANK2	10.09	15.00	4.39	−0.845688215
CXCL5	18.68	28.57	5.46	−0.866462528
MAS1	7.31	8.37	5.09	−0.918996442
FLJ10884	7.11	7.99	5.21	−0.922608325
SYT13	8.20	7.74	7.15	−0.930388821
DEFA1	17.12	13.97	9.33	−0.945454774
LOC254531	8.06	7.15	5.70	−0.951989998
DKFZP564O0523	9.67	11.20	4.31	−0.956606755
ZNF277	9.80	8.37	5.95	−0.957461038
COX6CP2	13.97	11.27	6.34	−0.962461413
TP73L	9.20	9.85	6.65	−0.962541835
PRO1496	11.83	9.38	4.89	−0.962971923
DKFZP434H132	6.52	7.00	4.30	−0.968953829
CCL27	21.35	24.51	4.44	−0.973907967
CCRL1	7.67	7.97	5.67	−0.979552762
GLRA2	16.63	15.51	12.76	−0.979704777
FLJ20045	14.96	12.76	5.22	−0.990893832
OR5V1	7.20	6.79	5.09	−0.994534383

We examined the top 30 most upregulated genes in each of the three doses of Meth used to stimulate astrocytes (Table 2). Surprisingly, the majority of the genes differed between the three doses. However, a few genes appeared among the 30 most upregulated genes by all conditions, compared to controls. These genes, which appear in Table 1 as italics, were the mitogen-activated protein kinase kinase 5 (MAP2K5), the G protein-coupled receptor 65 (GPR65), ectodysplasin A (ED1), the neuron navigator 3 (NAV3), and CXCL5, which were chosen for a deeper analysis of the behavior of genes in network with them and pathway changes resulting from Meth exposure.

**Table 2:** List of the 30 most upregulated genes by each one of the three doses of Meth utilized to stimulate astrocytes

Genes upregulated by Meth in primary astrocyte cultures						
	Dose 1 μM Meth	Fold change	Dose 10 μM Meth	Fold change	Dose 100 μM Meth	Fold change
1	SMG1	154.71	ZNF492	53.53	SLC12A1	124.70
2	LOC153077	49.55	KBTBD10	46.06	SMG1	120.32
3	*MAP2K5*	47.30	*MAP2K5*	40.66	CXCL11	72.41
4	POGZ	42.73	ADAM3A	39.21	TACR3	54.41
5	SSA2	37.28	*GPR65*	38.31	RBPMS	51.89
6	PHF3	30.59	KIAA0506	34.32	*MAP2K5*	51.74
7	*ED1*	28.51	EIF3S5	32.72	PTGER3	49.18
8	DKFZp547G183	27.90	FLJ21463	28.81	R29124_1	49.00
9	C13orf10	26.12	*CXCL5*	28.57	KIAA1579	48.75
10	ZNF198	25.51	PTGER3	27.56	PHF3	47.05
11	TNP2	23.46	FLJ11292	26.79	*NAV3*	43.44
12	NFAT5	23.00	SLC12A1	26.12	RGS13	42.76
13	CCL27	21.35	SAMSN1	25.74	SSA2	39.64
14	GAD2	19.96	*NAV3*	25.26	RIT2	39.33
15	XCL1	19.84	NSBP1	25.17	C14orf105	38.76
16	RW1	19.68	FLJ22595	25.09	RW1	35.53
17	*NAV3*	18.93	CCL27	24.51	IBTK	34.45
18	*CXCL5*	18.68	MLL4	20.74	CEACAM8	34.23
19	FLJ21463	18.35	FLJ13265	20.68	*ED1*	32.47
20	MDM2	17.42	MR1	20.62	FLJ21463	30.81
21	ERO1LB	17.38	KRTAP2-2	20.52	BLZF1	30.54
22	DEFA1	17.12	TAP2	20.47	ARTN	29.96
23	GLRA2	16.63	TRGC2	20.42	POGZ	29.02
24	DKFZp761K1824	16.25	ZNF198	20.32	CSHL1	28.22
25	FLJ11292	15.84	PC	19.67	*CXCL5*	27.58
26	MYO15B	15.81	ELL2	19.67	DLEU2	27.26
27	*GPR65*	15.67	TRIM29	19.48	SOX30	27.15
28	FLJ20045	14.96	TACR3	18.76	TAP2	27.07
29	ZNF407	14.94	*ED1*	18.13	*GPR65*	26.87
30	ADAM3A	13.97	CRISP1	17.94	TRGC2	26.81

The doses of Meth corresponded to low (1 μM), moderate (10 μM), and high (100 μM) Meth exposure. All genes in the list have been curated for significance as described in the Methods section. The genes in italics correspond to molecules that consistently appeared among the 30 upregulated genes in all three doses of Meth and, for this reason, were prioritized for validation.

**Fig. 2:**
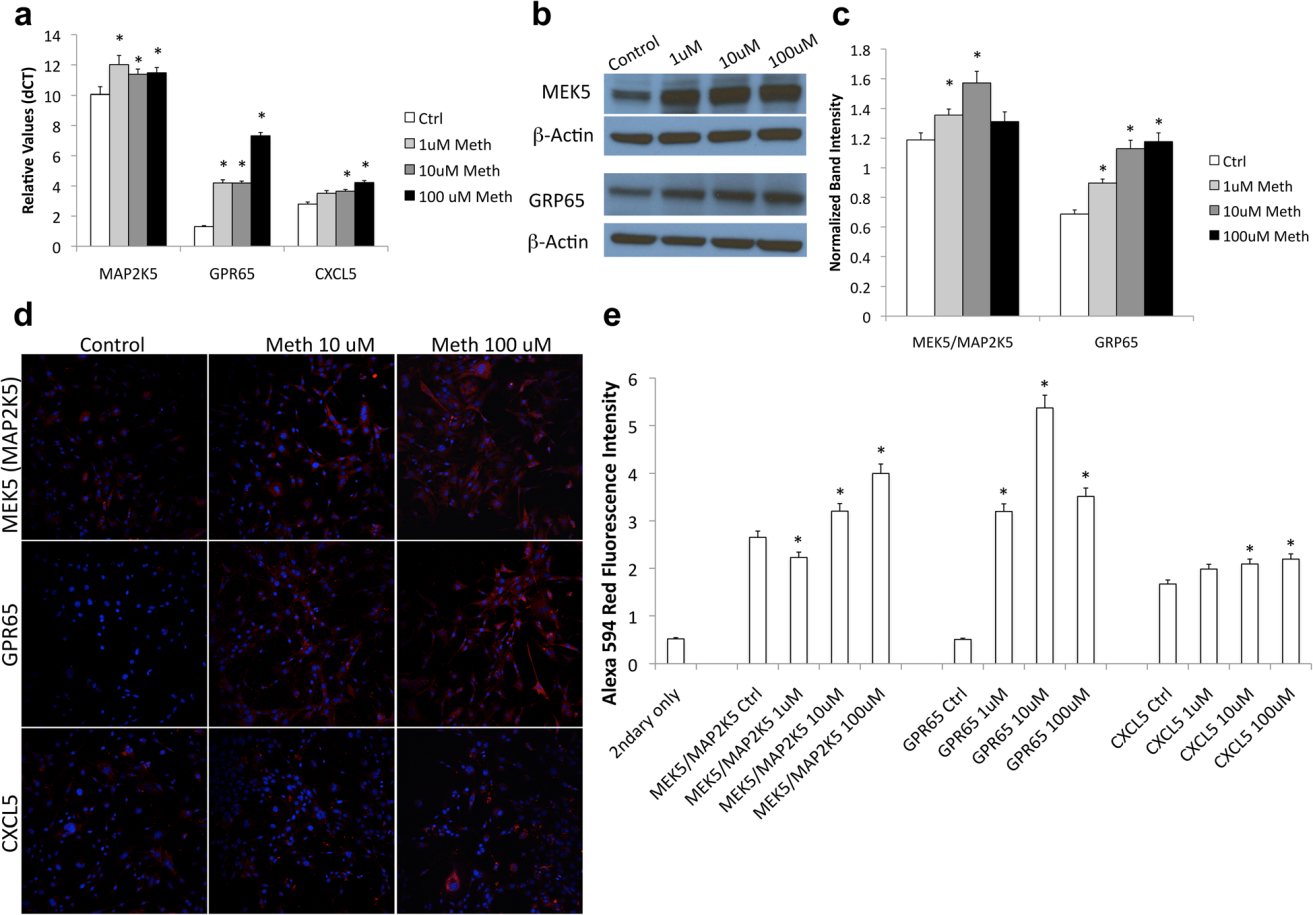
Validation of selected upregulated genes. **a** Transcriptional levels of MAP2K5, GRP65, and CXCL5 in astrocytes stimulated with 1, 10, and 100 μM of Meth for 24 h, examined by SyBrGreen qRT-PCR. Results are the Mean ± SEM of three independent experiments performed in duplicate. **b** Representative western blots for detection of MEK5, the protein encoded by MAP2K5 gene, GPR65, and normalizing b-actin, in protein extracts from astrocytes stimulated with 1, 10, and 100 μM of Meth for 24 h. **c** Normalized band intensity was calculated in ImageJ software (NIH). **d** Confocal imaging showing representative astrocytic cultures stained with specific antibodies for detection of MEK5, GPR65, and CXCL5 in 10 and 100 μM Meth treatments. **e** Fluorescence intensity of the expression of MEK5, GPR65, and CXCL5 was calculated in ImageJ (NIH) for cultures stimulated with 1, 10, and 100 μM, as well as controls. *p < 0.05 in one-way ANOVA followed by Bonferroni’s post hoc comparison against control conditions

MAP2K5, GPR65, and CXCL5 were further validated by PCR and also at the protein levels. The qPCR validation results confirmed the potential importance of these three molecules in the direct response of astrocytes to Meth (Fig. 2a). The PCRs were performed in the same samples used for gene array and also in two independent experiments. MAP2K5 and GRP65 were significantly upregulated by all three doses, and CXCL5 was significantly upregulated in 10 and 100 μM treatments (Fig. 2a). We confirmed the relevance of these findings at the protein level, using specific antibodies against MEK5, the protein transcribed by the MAP2K5 gene, and GRP65, by western blot (Fig. 2b, c). The enrichment of these proteins was also confirmed by imaging, using specific antibodies against MEK5, GRP65, and CXCL5 (Fig. 2c, d). The increase in these markers first identified using systems biology tools suggests the value of the approach and its power for the identification of changes in genes that depend on or are associated with MAP2K5, GRP65, and CXCL5. We again utilized systems biology to search for gene networks associated to these genes, as well as to ED1 and NAV3, exhibiting synchronic behaviors in response to Meth in astrocytes.

For the examination of the behavior of genes in network with MAP2K5, GPR65, CXCL5, ED1, and NAV3, we used GeneMania in Cytoscape, and for that, we focused on the 10 μM dose, which represents levels of drug reaching the brain in Meth users [23]. The analysis of the changes was conducted using a protocol utilized in our lab, to determine the behavior of genes associated to the ones we chose to prioritize, based on pathway, physical and genetic interactions, shared protein domains, or coexpression, in order to predict molecular networks with which astrocytes might respond to acute drug abuse. Using GeneMania and JActiveModules in Cytoscape [24–27], we identified such gene node clusters. The highest score node contained 141 genes, which were all upregulated, and which clustered with a coefficient of 0.174, suggesting that Meth has a strong effect on astrocytic gene networks. Ninety of those genes (63.8%) showed multi-edged node pairs, suggesting a strong interaction between molecular changes and processes triggered by Meth in astrocytes (Additional file 1: Figure S1). MAP2K5, GPR65, and ED1 (EDA or the rat homolog of CD68), which were consistently among the 30 genes most upregulated by all three doses of Meth (Table 2—italic letters), were also represented in this large gene cluster. A literature examination suggests that these genes could be a link between the acute response of astrocytes to Meth and the potential development of CNS alterations. For instance, ED1 is a microglial marker but it can be found expressed on tumoral astrocytes [28]. Interestingly, MAP2K5 is also a characteristic of tumorigenesis [29]. GPR65, on the other hand, is a proton- and acid-sensing G-protein receptor that plays an important role in cell survival and is also known as TDAG8 [30, 31].

**Fig. 3:**
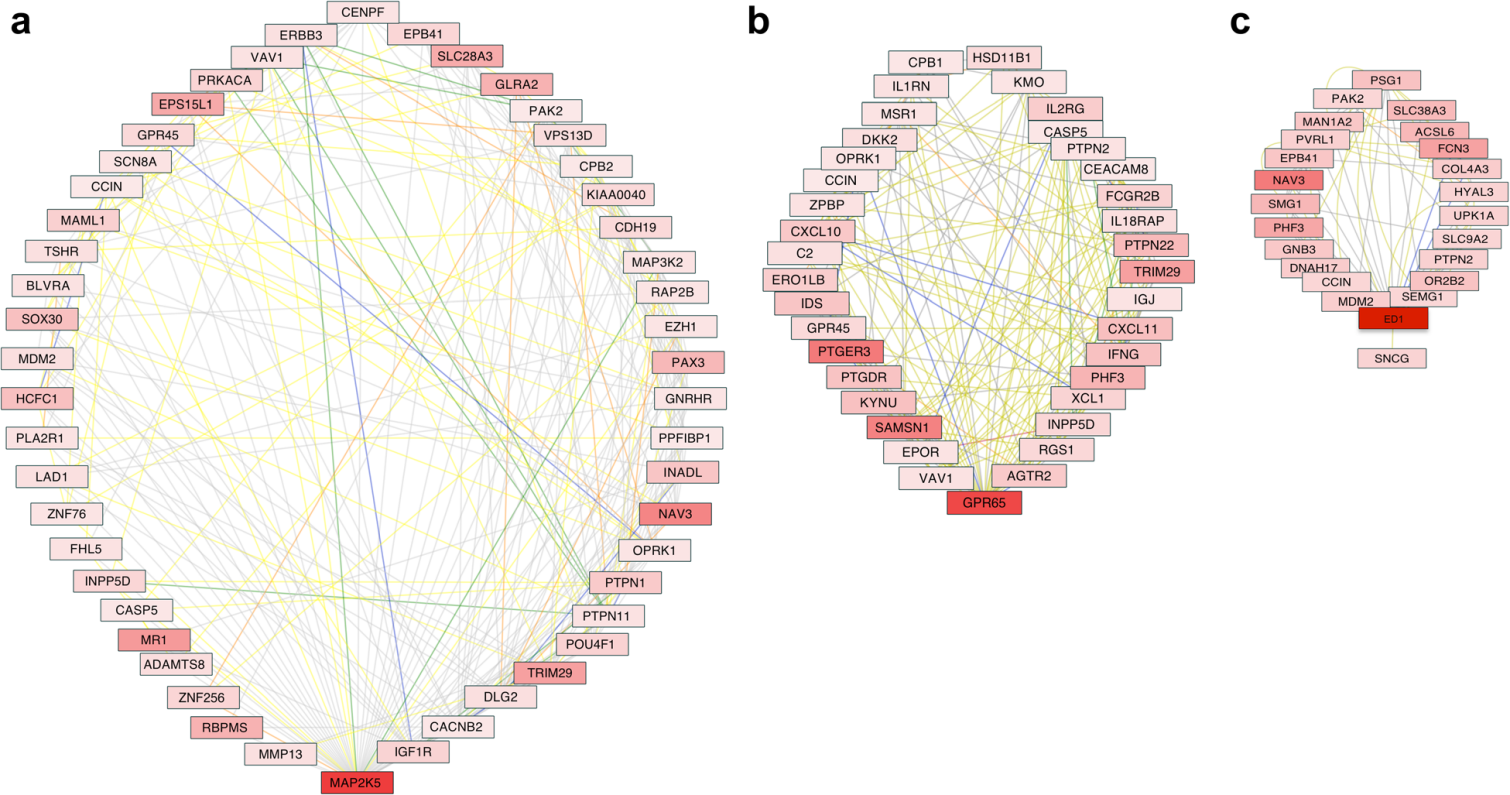
Connectivity of genes that were upregulated in astrocytes by Meth. JActiveModules was applied to the whole data set, using a significant (*p* < =0.05) minimum of a 4-fold upregulation, in genes identified in astrocytes that were treated with 10 μM Meth for 24 h, for identification of hotspots. This approach identified a (**a**) cluster of MAP2K5-first neighbor genes, a (**b**) cluster of GPR65-first neighbor genes, and a (**c**) cluster of ED1-first neighbor genes. Shades of red represent the level of upregulation of each gene, where light red is less upregulated and darker red is more upregulated. Genes that are connected by a green line are in the same pathway, whereas orange lines connect genes that are colocalized, yellow lines mean coexpression, gray lines mean genetic interactions, blue lines mean shared protein domains, and red lines mean physical interactions

We examined subfamilies of genes assembled as child nodules, by connecting first neighbors of MAP2K5 (Fig. 3a) and GPR65 (Fig. 3b), which led to subnetworks respectively assigned to neuronal support and inflammation. For instance, the genes that appeared in connection with MAP2K5 (Fig. 3a) were annotated to MAPK signaling (*p* = 0.0022), gap junction (*p* = 0.0051), the GnRH signaling pathway (*p* = 0.0062), and also neuroactive ligand-receptor interactions (*p* = 0.008), suggesting association to neurological outcomes. The genes connected to GPR65 (Fig. 3b) were associated to pathways involving cytokine-cytokine interaction (*p* = 0.00027), chemokine signaling pathway (*p* = 0.04), B cell receptor signaling pathway (*p* = 0.003), Fc-gamma R-mediated phagocytosis (*p* = 0.0048), and systemic lupus erythematous (*p* = 0.0052), suggesting a role in potential inflammatory outcomes.

ED1 was not represented in either one of these subnetworks, but a cluster analysis centered on first neighbors of this gene (Fig. 3c), resulted in a group of genes functionally assigned as glycoproteins (*p* = 0.0018), and functionally annotated to immune response (*p* = 0.029) and cell adhesion (*p* = 0.03).

**Fig. 4:**
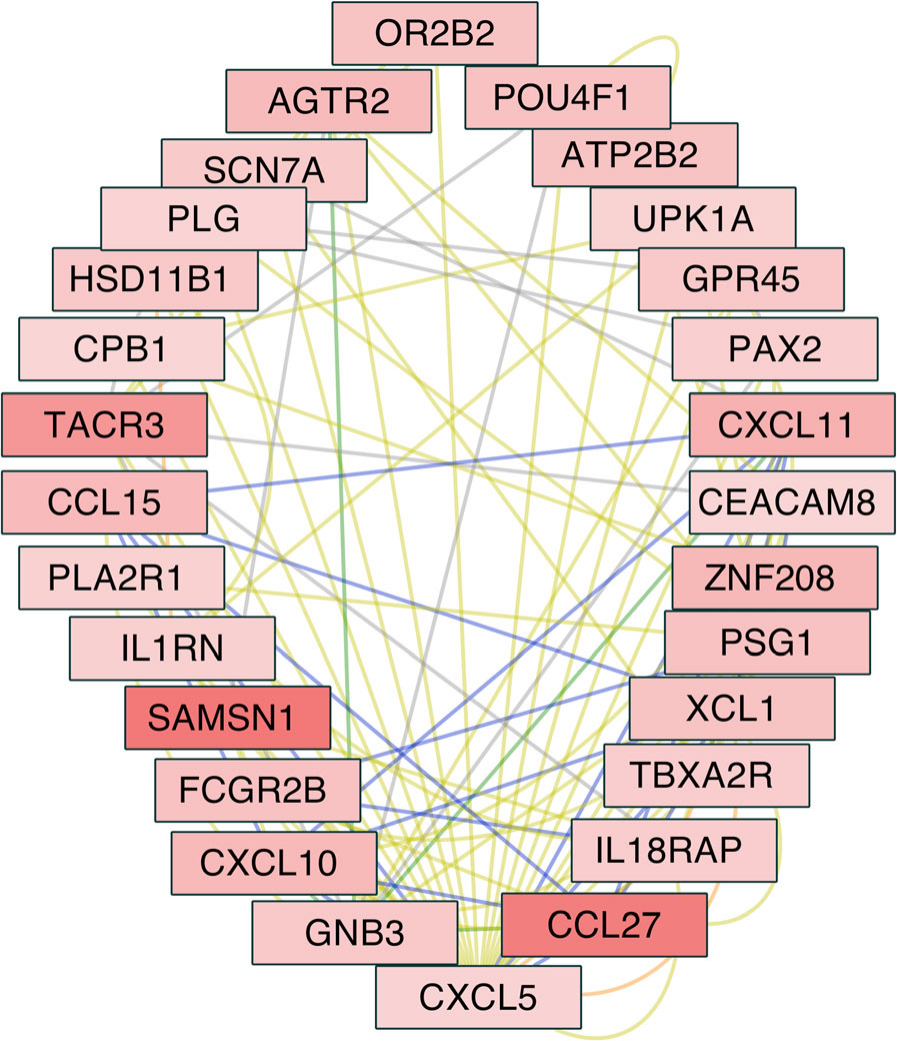
CXCL5-centered gene network upregulated in astrocytes in response to Meth exposure. *Shades of red* represent the level of upregulation of each gene, where *light red* is less upregulated and *darker red* is more upregulated. Genes that are connected by a *green line* are in the same pathway, whereas *orange lines* connect genes that are colocalized, *yellow lines* mean coexpression, *gray lines* mean genetic interactions, *blue lines* mean shared protein domains, and *red lines* mean physical interactions

Other genes among the 30 most upregulated ones by all three doses were NAV3 and CXCL5 (Table 2). NAV3 was represented within subnetworks associated to GPR65 (Fig. 3b), as well as to ED1 (Fig. 3c). CXCL5, on the other hand, segregated on a network (Fig. 4) that was heavily associated to chemokine signaling pathway (*p* = 8.7E−7), cytokine-cytokine receptor interaction (*p* = 6E−5), neuroactive ligand-receptor interaction (*p* = 0.0037), and calcium signaling pathways (*p* = 0.01).

**Fig. 5:**
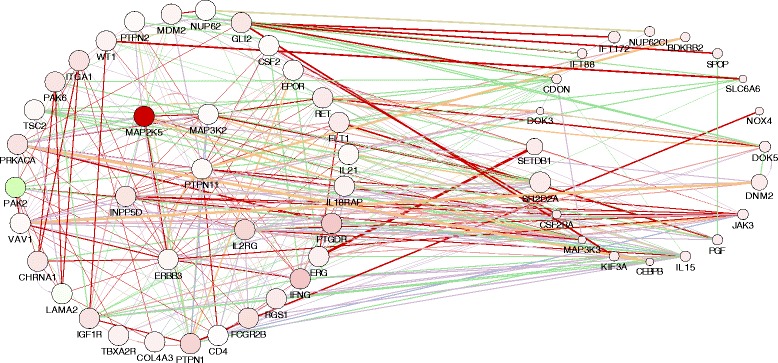
MAP2K5-centered gene clusters exclusively based on pathway and physical interactions. The data was filtered by eliminating colocalization, coexpression, genetic interactions, and shared protein domains from the clustering criteria, but maintaining physical and pathway interactions. *Shades of red* represent the level of upregulation, and *green* represents downregulation of each gene. *Red lines* represent pathway associations, and *green lines* represent associations based on protein physical interactions. A group attributes layout was applied to discriminate *p* values and scores. Scores can be also identified by the size of individual nodes. Genes grouped on the left had lower *p* values, and highest scores than genes on the right. Genes in the center of the left group were the ones with highest scores, or highest number of connectors

We further dissected the highest score node, to produce subnetworks that were derived by the introduction of a display restriction connecting genes only through pathway and physical associations. This restrictive approach generated one network (Fig. 5), in which MAP2K5 appeared as the strongest upregulated gene, in correlation with other genes described in neurological processes, metabolism, and inflammation (Fig. 5). Importantly, as a control, the cortical astrocyte marker ErbB3 [32] was represented in this network.

**Fig. 6:**
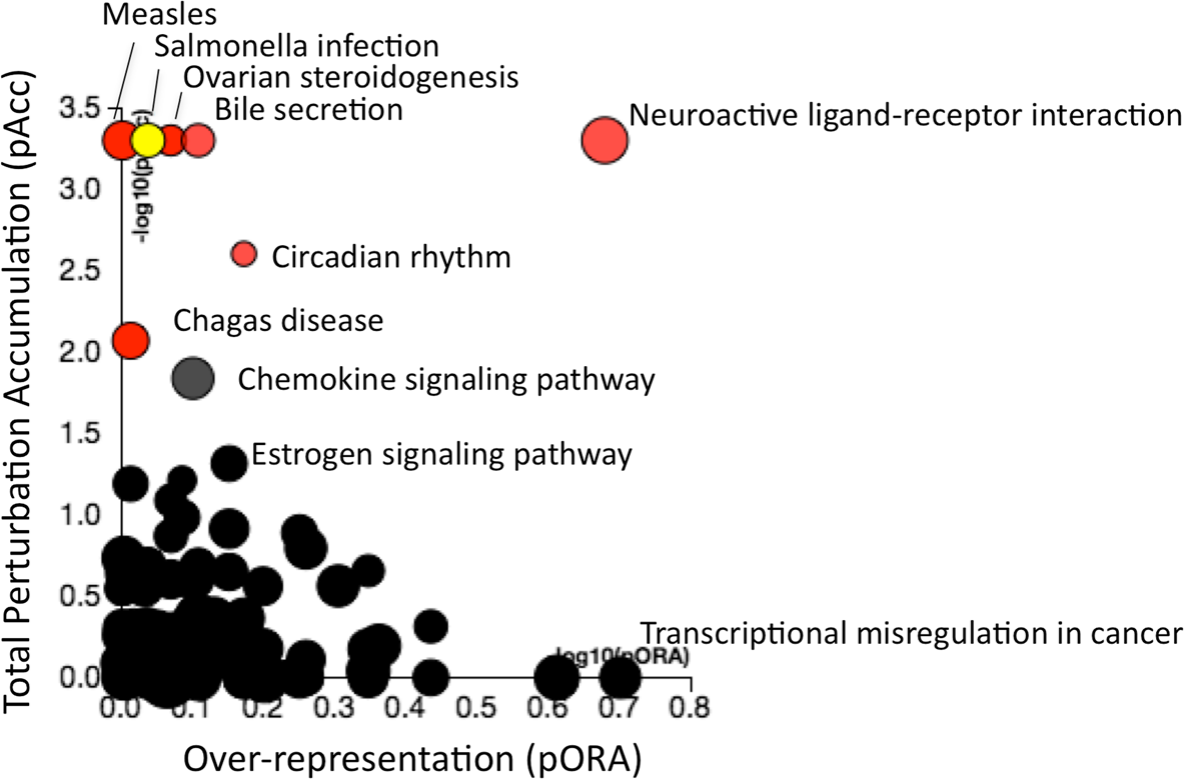
Pathway enrichment analysis on genes upregulated by Meth treatment of astrocytes. iPathwayGuide derived analysis showing molecular pathways segregated according to total perturbation accumulation (pACC) and gene overrepresentation within pathways (pORA). *Red*/*Yellow dots* represent pathways modified with a significant *p* value

We performed pathway enrichment analysis on the set of genes that were upregulated by Meth in astrocytes, using the DAVID Bioinformatics Database (KEGG_PATHWAY) (Table 3) and the iPathwayGuide (Fig. 6). We found that Meth treatment on astrocytes caused an important enrichment of genes that are relevant in neuroactive ligand-receptor interactions, immunity, and metabolic outcomes, as shown in Table 3.

**Table 3:** Statistically significant pathways that are disturbed in astrocytes following Meth exposure

Pathway term	*p* value	Benjamini
Neuroactive ligand-receptor interaction	0.00053	0.044
Cytokine-cytokine receptor interaction	0.00079	0.035
Steroid hormone biosynthesis	0.0012	0.037
Androgen and estrogen metabolism	0.0025	0.051
Calcium signaling pathway	0.0032	0.051
Chemokine signaling pathway	0.0042	0.055
GnRH signaling pathway	0.0082	0.077
Glioma	0.0092	0.075

DAVID was utilized for identification of pathways with important gene representation in changes induced by Meth

Further analysis was conducted to examine the number of gene perturbation accumulation (pACC) versus overrepresentation *p* value (pORA) within pathways (Fig. 6) and that also indicated that CNS- and pathology-relevant genes were strongly represented. For instance, genes involved in neuroactive ligand-receptor interaction (*p* = 0.001) and circadian rhythm (*p* = 0.012) led to an important contribution to changes induced by Meth in astrocytes. A substantial, but not statistically significant, representation of genes involved in chemokine signaling pathways was also observed (*p* = 0.056). Interestingly, Meth also triggered genes that are involved in the resistance to infections (Fig. 6). The strong representation of pathways that may be involved in neurological outcomes prompted an analysis of individual genes.

**Fig. 7:**
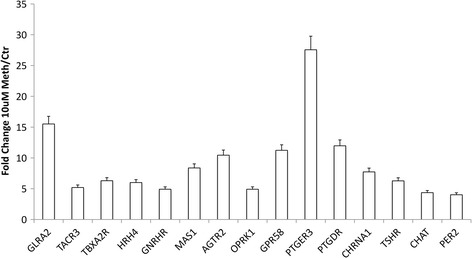
Genes mapped to the neuroactive ligand-receptor pathway, identified by gene array in astrocytes that were treated with Meth. Data corresponds to significant fold change in 10 μM Meth-treated astrocytes compared to vehicle-treated controls in the gene expression array

Of the genes annotated to the neuroactive ligand-receptor interaction pathway (Fig. 7), a few were interesting for being associated to neurological disorders in other systems, for instance, the Period Circadian Clock 2 (PER2) [33, 34]. Others have been also described to have essential inflammatory roles, for instance, prostanoid receptors, such as the prostaglandin E receptor 3 (PTGER3), the thromboxane A2 receptor (TBXA2R), and the prostaglandin D2 receptor (PTGDR), which play important roles in inflammatory reactions and hyperalgesia [35].

The other genes upregulated by Meth treatment of astrocytes that could be mentioned for their potential connection with CNS-related syndromes were the following: the inositol 1,4,5-triphosphate receptor, type 1 (ITPR1, 9.24-fold upregulation) is involved in spinocerebellar ataxia [36]; the cold autoinflammatory syndrome 1 (CIAS1, 10.94-fold) in cryopirin-associated periodic syndrome [37]; the RET proto-oncogene (7.4-fold) in congenital central hypoventilation syndrome [38, 39]; PER2 (4-fold) in familial advanced sleep phase syndrome [40]; nucleoporin 62 kDa (NUP62, 4.72-fold) in infantile bilateral striatal necrosis [41]; ubiquitin-activating E1 (UBE1,10–45-fold) [42] and ankyrin repeat domain 2 (stretch responsive muscle, ANKRD2, 9.06-fold) in paralytic syndromes and cerebral palsy; interleukin 1 receptor antagonist (IL1RN, 6.43-fold), the interleukin 2 receptor common gamma chain (IL2RG, 10.02-fold), the ATP-transporter 2 (TAP2, 20.47-fold), PTGDR (11.97-fold), PTGER3 (27.56-fold), TBXA2R (6.3-fold), and the Complement Component 2 (C2, 5.8-fold) in CNS inflammation; the AarF domain containing kinase 4 (ADCK4, 11.04-fold) in coenzyme Q deficiency [43]; the zinc finger protein 41 (ZNF41, 5.39-fold) and the angiotensin II receptor, type 2 (AGTR2, 10.44-fold) in fragile X syndrome, cerebral ataxia, mental retardation, and disequilibrium syndrome [44, 45]; laminin, alpha 2 (LAMA2, 4.31-fold) in congenital muscular dystrophies and control of the blood-brain barrier [46]; and the cholinergic receptor, nicotinic, alpha 1 (CHRNA1, 7.73-fold) involved in congenital myasthenic syndrome and considered as an important potential drug target [47].

**Fig. 8:**
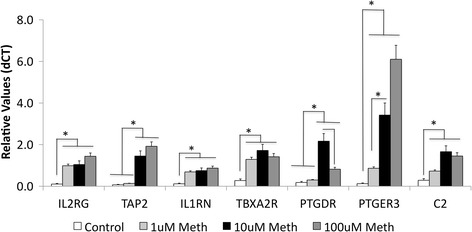
Transcriptional validation of inflammatory gene changes induced by Meth in astrocytes. Gene expression was examined by SyBrGreen qRT-PCR on mRNA extracts from primary astrocytes treated with 1 μM, 10 μM, and 100 μM of Meth, as well as controls, upon GAPDH normalization. Results are from one representative experiment, confirmed by two independent biological replicates. *p < 0.05 in one-way ANOVA followed by Bonferroni’s post hoc test, in comparisons indicated by *lines*

Using qRT-PCR, we confirmed the transcriptional upregulation of several of these genes, prioritized by their presence in the two most strongly represented pathways, the neuroactive-ligand and the cytokine-cytokine receptor interaction pathways (Table 4), in all three doses of Meth in vitro. These genes were the IL1RN, IL2RG, as well as the prostanoid receptors PTGDR, PTGER3, and TBXA2R (Fig. 8 and Table 4). These genes play important functions in inflammatory processes in the brain and elsewhere. We also examined other upregulated genes that have been described as molecules that could potentially influence the immune environment in the CNS, TAP2, and C2 (Fig. 8). All these validated genes have been suggested to play important roles in the CNS and neurological disorders [23, 48–53]. Our validation by PCR confirmed that the Meth treatment has the capacity to directly stimulate the upregulation of these genes, which have an involvement in inflammatory processes in the brain. Among them, TAP2 and PTGDR were upregulated by 10 and 100 μM Meth but not by 1 μM, while the other genes were transcriptionally increased by all the doses, validating the gene array data.

**Table 4:** Genes that are present in the highest represented pathways upregulated by Meth in astrocytes

Genes in the neuroactive ligand-receptor Interaction Pathway	Fold change in 10 μM/control	Genes in cytokine-cytokine receptor interaction pathway	Fold change in 10 μM/control
PTGER3	27.56	CCL27	27.51
GLRA2	15.51	CXCL11	12.83
PTGDR	11.97	IFNG	12.46
AGTR2	10.44	CXCL10	10.82
MAS1	8.37	CCL15	10.54
GH2	7.37	IL2RG	10.02
TBXA2R	6.30	XCL1	8.61
TSHR	6.27	GH2	7.37
HRH4	6.00	IL1RN	6.42
TACR3	5.19	IL18RAP	6.14
OPRK1	4.91	CXCL5	28.57
GNRHR	4.91	IL21	5.35
PLG	4.70	EPOR	5.05
		CSF2	5.04

DAVID was utilized for identification of pathways with important gene representation in changes induced by Meth and the genes within pathways that change as a result of Meth exposure on astrocytes

**Fig. 9:**
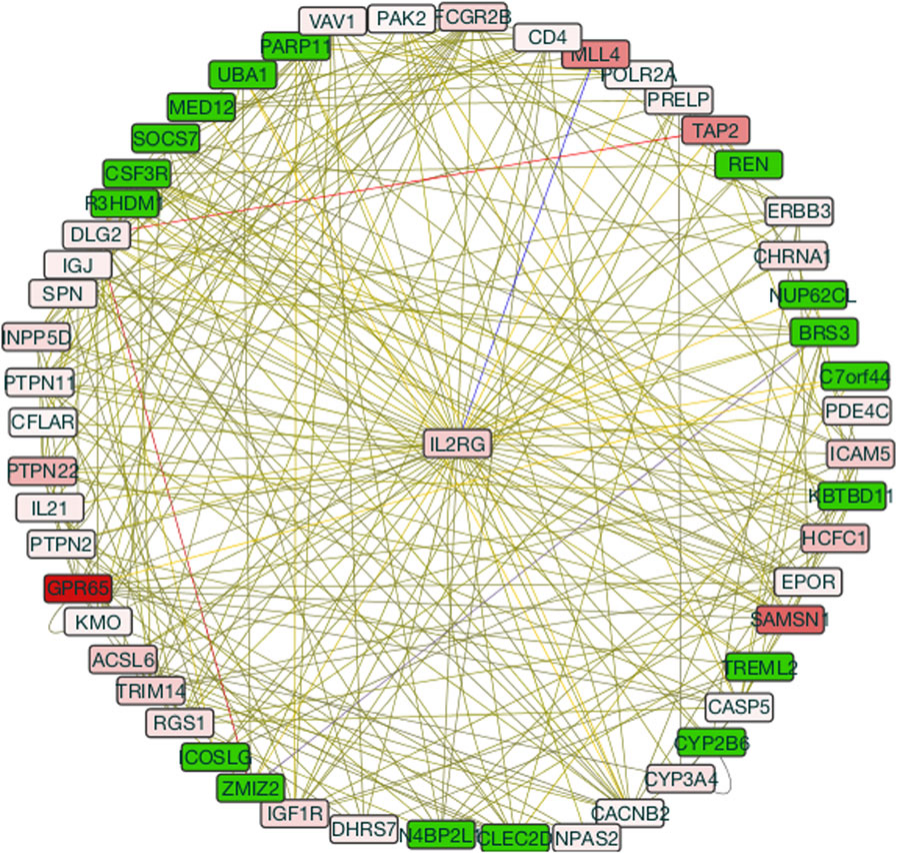
IL2RG-centered gene subnetwork. Network shows connected genes based on pathway (*red lines*) and physical interactions (*brown*). The data was filtered by eliminating colocalization, coexpression, genetic interactions, and shared protein domains from the clustering criteria, but maintaining physical and pathway interactions. *Shades of red* represent the level of upregulation, and *green* represents downregulation of each gene

Although our analysis of changes in astrocytic gene expression was focused on enrichments, a few of the genes highlighted in our study have showed network connections with genes downregulated by the Meth treatment. The increase in MAP2K5, for instance, was associated with the decrease on serine/threonine protein kinase 2 (PAK2) (0.6-fold, *p* = 0.041) (Fig. 5). Other molecule that showed several downregulated connectors was IL2RG (Fig. 9). A gene cluster centered on IL2RG was associated with several components of the Jak-Stat signaling pathway (*p* = 0.0002, Benjamini = 0.002), primary immunodeficiency (*p* = 0.008, Benjamini = 0.03), and cytokine-cytokine receptor interaction (*p* = 0.006, Benjamini = 0.06) pathways. Of the genes in the IL2RG interactive subnetwork, several were significantly downregulated, such as the inducible T cell costimulator ligand (ICOSLG) (0.3-fold, *p* = 0.006) or the cytochrome P450 family member CYP2B6 (0.1-fold, *p* = 0.0012), suggesting that the upregulated genes may interfere with, or become affected by, transcriptional suppressions. The role of the downregulated genes in the development of astrocytic changes caused by Meth must be examined in the future.

## Discussion

We examined the hypothesis that astrocytes respond to Meth exposure by developing gene expression signatures and upregulation of genes that may participate in pathogenesis. For that, we used an in vitro approach, where primary cortical astrocyte cultures were directly exposed to Meth for 24 h. Our system-wide approach combined experimental and computational methods to systematically identify and integrate the important characteristics of astrocytes following the direct exposure of Meth, without taking into account downregulated genes. This approach was chosen in order to address a gap in the knowledge of dopamine-independent effects of drug abuse, which may play an important role in the development of response patterns in glial cells. With a focus on significantly upregulated genes, we identified signature patterns that were validated by qPCR and by changes at the protein level. A review of the literature revealed that several of these markers could have implications to CNS pathogenesis.

The validated changes in MAP2K5 (MEK5) levels and the detection of changes in many genes associated with this kinase were particularly remarkable and indicated a pathway of interest. MAP2K5 is a component of the MAPK family intracellular signaling pathway in the brain. In the context on cell-cell communication, it is highly responsive to extracellular growth factors such as brain-derived neurotrophic factor (BDNF), nerve growth factor (NGF), insulin-like growth factor 2 (IGF2) [54], granulocyte colony-stimulating factor [55], and epidermal growth factor [56]. MAP2K5 is particularly responsive to oxidative stress, for instance, in muscle differentiation where it activates ERK5 [57]. In the brain, its upregulation could play a role in neuroprotection of dopaminergic neurons [58], suggesting that the acute response of astrocytes to drug abuse could provide survival signals. On the other hand, in isolated astrocyte cultures, the MAP2K5 gene clusters with other genes that suffered similar changes, which have been described to be involved in CNS disorders. One of these genes is LAMA2, which is associated with muscular dystrophies and with the blood-brain barrier control [46]. LAMA2 also regulates other genes, such as the ladybird homeobox corepressor 1 (LBXCOR1), which is a corepressor of transcription playing a role in GABAergic phenotype of interneurons in some areas of the brain, associated for instance to the susceptibility to restless legs syndrome [59]. Another one of these genes is MDM2, which is described as an important regulator of tumorgenesis in astroglioma models [60].

GPR65, which was also validated, is also known as T cell death-associated gene 8 (TDAG8), an acidosis-sensing molecule [61, 62] and that has been described in association with susceptibility to autoimmunity, including in the CNS. Given that one of the adverse effects of Meth abuse is metabolic acidosis [63], this finding could be of relevance. On the other hand, GPR65 is a negative regulator of inflammation [64, 65]. Its upregulation may partially protect the brain environment in the response to acute Meth. In astrocytes exposed to Meth, CXCL5 was one of the genes highly upregulated by all Meth doses, with a strong connection to inflammatory pathways that can potentially lead to important changes in the context of the brain environment [66]. Importantly, CXCL5 has been reported as a potential marker of ischemic brain injury [67]. Conversely, the most enriched molecular pathways corresponded to Neuroactive ligand-receptor interactions and cytokine-cytokine receptor interactions, which respectively include MAP2K5 and GPR65, as well as CXCL5 genes.

We found that Meth caused the upregulation of several prostanoid receptors, including TBXA2R, PTGDR, and PTGER3, on astrocytes. These characteristics in the context of the brain could contribute to the inflammatory pathophysiology seen in the CNS of Meth abusers. These targets were validated by quantitative real-time polymerase chain reaction (qRT-PCR). Interestingly, the expression of PTGER3 was particularly sensitive to Meth, in a dose-dependent manner. In the context of pathology, PTGER3 has been found to be upregulated in aged patients with schizophrenia, suggesting the participation of the eicosanoid signaling in mental disorders [68]. In addition, the prostanoid receptors could affect major signaling pathways, such as the MAP kinase pathway [69], providing a potential link between the inflammatory and neuroactive aspects of the astrocytic acute response to the drug.

There was an intriguing induction of circadian rhythm-associated molecules in astrocytes that were treated with Meth. This supports findings in the literature where mammalian astrocytes display circadian functional profiles, particularly regarding the expression of the clock genes such as PER2, as well as regarding ATP release [33, 34]. It remains to be determined whether the expression of this gene specifically in astrocytes can play a role in vivo, since it has been shown that PER2 does modify circadian sleep cycle during sleep disruptions [70] and also in substance abuse [71, 72].

The genes that promote inflammation are of particular interest, because when applied to the context of the brain, they could play a role both in neuroprotection and neurodegeneration [73, 74]. In Meth abuse, it is known that neuroinflammation plays a critical role in the development of neurological decline [75–78]. Our results indicate that the acute, direct response of astrocytes to the drug could contribute to the inflammatory pathogenesis. Therefore, the genes with pro-inflammatory roles in the two most enriched pathways, which were the neuroactive ligand-receptor and the cytokine-cytokine receptor interactions, were prioritized for validation. These genes were the IL2RG, TAP2, and IL1RN, in addition to the prostanoid receptors. The ability of Meth to upregulate them was confirmed by qRT-PCR. The induction of IL2RG by Meth in astrocytes could be a factor modulating phenotype of other glial cells in vivo, as previously described by us in the context of HIV/Meth comorbidities [23]. TAP2, on the other hand, is a molecule that is involved in the expression of class I major histocompatibility complex molecules, which affects antigen presentation [79–82]. The role of the class I-mediated cytotoxic response in the HIV and in the HIV/Meth comorbidities has also been described by us [80, 81, 83, 84]. We also validated the upregulation of IL1RN by Meth, which could contribute to controlling astrocytosis, as suggested in the prion disease model [85]. IL1RN has been also suggested to participate in the regulation of glutamate uptake by astrocytes [86]. These genes are therefore important in a context of cellular interactions in the brain, and our results demonstrate that the acute exposure to the drug can cause their upregulation in a direct and dopamine-independent manner.

Several of the genes acutely induced by Meth in astrocytes have been described in association with neurological and inflammatory functions and with CNS disorders that are linked to motor dysfunction [87, 88]. Interestingly, astrocytes have been previously suggested as important cellular targets in CNS motor disorders [87, 88], although specific molecular targets are not clear. In the context of Meth abuse, movement disorders are very common sequels and comorbidities [89]. On the other hand, the genes upregulated by Meth in astrocytes also suggest a potential acute neuroprotective response. Interestingly, the use of low doses of Meth has been previously suggested to be beneficial immediately following severe traumatic brain injury [90, 91]. However, whether the character of the astrocytic response favors protection or neuronal damage and disorder, acutely or during chronic exposure remains to be investigated in vivo.

An important aspect of some upregulated genes examined here was their connection with downregulated network components. That was the case for genes associated with the increased expression of MAP2K5 and also IL2RG. The molecules in connection with the expression of IL2RG were of particular interest, given the role of the IL2RG system components in brain inflammatory outcomes and modulation of microglial phenotypes [92]. The IL2RG subnetwork was associated with the increase of important pro-inflammatory molecules such as IL21 [93] but also with the decrease of molecules such as the inducible T cell costimulator ligand (ICOSLG), which is a pattern recognition element that may impact immune response [94], or the cytochrome P450 family member CYP2B6, which plays a role in mood disorders and depression [95]. The interaction between genes that are up- and downmodulated suggests the importance of extending the analysis to genes that are decreased by Meth, for understanding the full spectrum and implications of changes caused by the direct exposure of astrocytes to that drug.

Our data analysis has suggested that acute Meth exposure drives the development of response patterns in astrocytes that may cause these cells to play essential roles in vivo, through dopamine-independent mechanisms. There are limitations in the single-cell type system, as in vivo factors derived from the other cells may further modulate the effects of Meth on astrocytes, and the response patterns may not be exclusively a result of direct stimulation. Therefore, given that the genes that are increased directly by Meth can play critical roles influencing the brain cell network, the changes in astrocyte gene expression do need further examination, using in vivo models of drug abuse. The focus on upregulated genes may limit the interpretation of the data but may accelerate pre-clinical approaches, since upregulated molecules are favored as targets for therapeutics. The gene signatures identified among upregulated genes are highly relevant to inflammation and to CNS disorders but also suggest that Meth may trigger neuroprotection pathways. These deviations in the astrocytic response pattern may point to important targets to be further investigated, for preventing and ultimately reverting deleterious consequences and neurological sequels of drug abuse.

## Conclusions

Astrocytes modify their molecular signatures in response to direct exposure to methamphetamine. By applying a systems biology analysis approach with a focus on upregulated molecular markers, we have identified overexpressed gene networks represented by genes of an inflammatory and immune nature and that are implicated in neuroactive ligand-receptor interactions. MAP2K5, GPR65, and CXCL5 were molecules situated in the core of the highest score gene networks and were linked to markers associated with both neuroprotection and neuropathology. We have validated several targets and discussed their potential association with human neurological disease. Further in vivo studies are necessary to examine the role of these gene networks in drug abuse pathogenesis and their potential as biomarkers.

## Additional file

Additional file 1: Visualization of gene changes and networks for identifying over-expression patterns and for initializing the analysis of astrocytic gene network behaviors upon Meth exposure. Genes were connected based on pathway, physical and genetic interactions, shared protein domains, or co-expression, using GeneMania and JActiveModules in Cytoscape platform. Highest score nodes were grouped by circular layout. (PNG 6917 kb)

## Abbreviations

ADCK4: AarF domain containing kinase 4
AGTR2: Angiotensin II receptor, type 2
ANKRD2: Ankyrin repeat domain 2
ANOVA: Analysis of variance
BBB: Blood-brain barrier
BDNF: Brain-derived neurotrophic factor
C2: Complement component 2
CHRNA1: Cholinergic receptor, nicotinic, alpha 1
CIAS1: Cold autoinflammatory syndrome 1
CNS: Central nervous system
CXCL5: C-X-C chemokine ligand 5
DAVID: Database for annotation, visualization, and integrated discovery
ED1: Ectodysplasin A
FDR_BH: False discovery rate by the Benjamini-Hochberg correction
GPR65: G protein-coupled receptor 65
IGF2: Insulin-like growth factor 2
IL1RN: Interleukin 1 receptor antagonist
IL2RG: interleukin 2 receptor common gamma chain
ITPR1: Inositol 1,4,5-triphosphate receptor, type 1
KEGG: Kyoto Encyclopedia of Genes and Genomes
LAMA2: Laminin, alpha 2 (, 4.31-fold)
LBXCOR1: Ladybird homeobox corepressor 1
MA2K5: Mitogen-activated protein kinase kinase 5
Max LS: Maximum least-squares
Meth: Methamphetamine
NAV3: Neuron navigator 3
NGF: Nerve growth factor
pACC: Gene perturbation accumulation
PER2: Period Circadian Clock 2
pORA: Overrepresentation *p* value
PTGDR: Prostaglandin D2 receptor
PTGER3: Prostaglandin E receptor 3
TAP2: ATP-transporter 2
TBXA2R: Thromboxane A2 receptor
UBE1: Ubiquitin-activating E1
ZNF41: Zinc finger protein 41
