# Supplementary figures and images for: Astrocyte-specific overexpressed gene signatures in response to methamphetamine exposure in vitro
Source: J Neuroinflammation. 2017 Mar 9;14:49. doi: 10.1186/s12974-017-0825-6 (PMC5345234; doi:10.1186/s12974-017-0825-6)

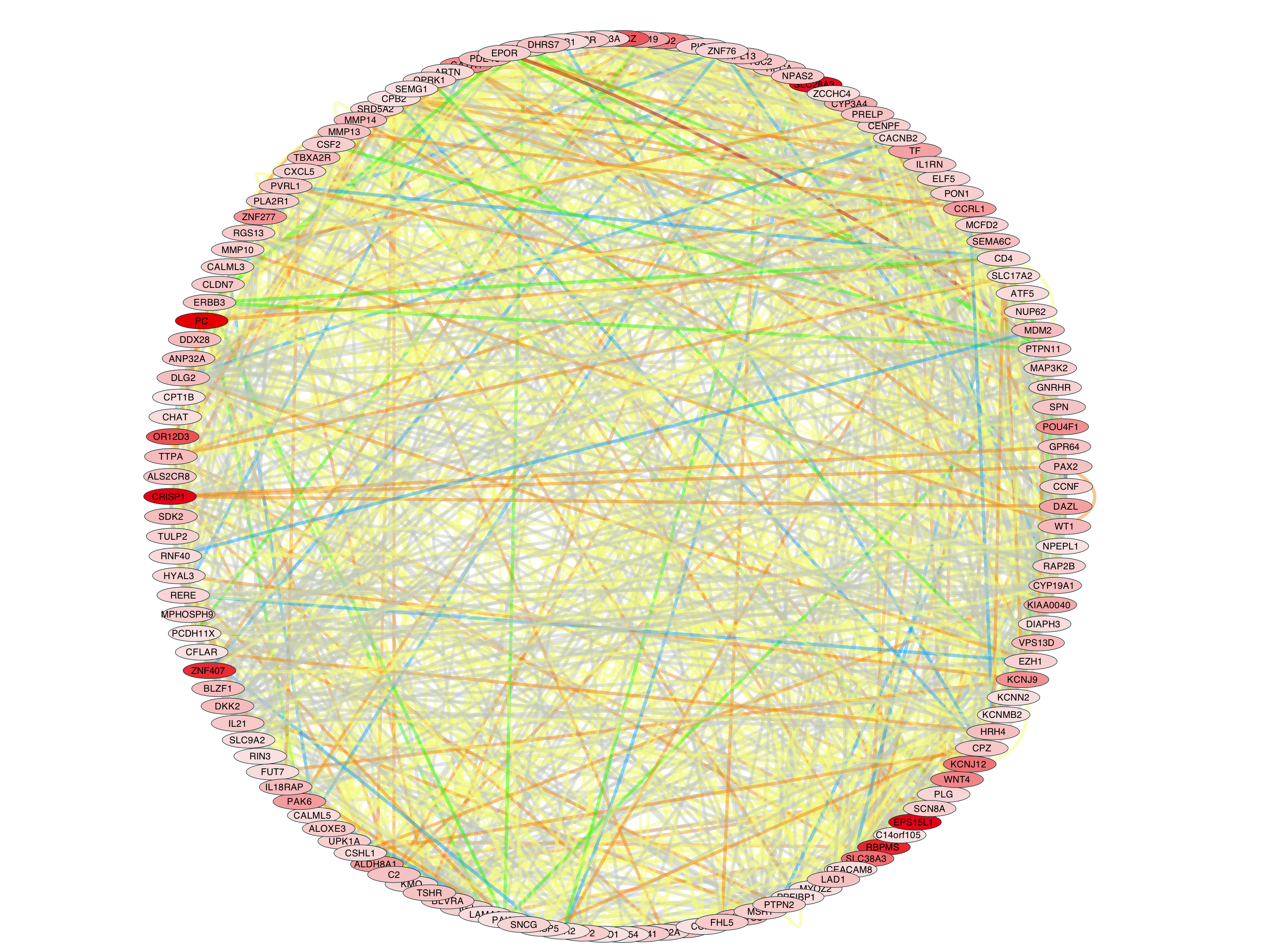

Supplement: Additional file 1: — Visualization of gene changes and networks for identifying over-expression patterns and for initializing the analysis of astrocytic gene network behaviors upon Meth exposure. Genes were connected based on pathway, physical and genetic interactions, shared protein domains, or co-expression, using GeneMania and JActiveModules in Cytoscape platform. Highest score nodes were grouped by circular layout. (PNG 6917 kb) [file 12974_2017_825_MOESM1_ESM.png]
